# The influence of oral health on sports performance: an interdisciplinary perspective

**DOI:** 10.1038/s41415-025-9348-1

**Published:** 2026-02-27

**Authors:** Karsten Hollander, Inbar Eshkol-Yogev, Astrid Zech, Jacopo Buti, Ian Needleman

**Affiliations:** 564920849429468781620 Institute of Interdisciplinary Exercise Science and Sports Medicine, MSH Medical School Hamburg, Germany; 737997554675180993619 Unit of Periodontology, Eastman Dental Institute, UCL, London, United Kingdom; 692008140901082029562 Department of Human Movement Science and Exercise Physiology, University of Hamburg, Germany; 545635107157382758571 Unit of Periodontology, Eastman Dental Institute, UCL, London, UK; Centre for Oral Health and Performance, Eastman Dental Institute, UCL, London, United Kingdom

## Abstract

Sports performance is a multifactorial concept determined by the interplay of physical, technical, tactical, and psychological factors. While its measurement is straightforward in metrical sports (e.g., athletics or swimming), it is more complex in team or aesthetic disciplines. In recent years, the role of oral health has gained attention as a potentially modifiable factor influencing athletic performance. This paper provides a comprehensive overview of how sports performance is defined and measured – particularly in explosive and endurance sports – and explores the impact of oral health on performance outcomes. Evidence from systematic reviews and observational studies shows consistent associations between poor oral health (e.g., periodontal disease, caries, malocclusion) and reduced objective performance metrics such as V̇O_2_ max, power output, speed, and agility, as well as self-reported reductions in training capacity and competitive performance. Potential mechanisms include systemic inflammation, impaired nutrition, altered microbiomes, psychological burden, and altered sensorimotor control. These pathways highlight the relevance of oral health in both recovery and performance optimisation. Future research in sports dentistry should adopt an interdisciplinary approach, using validated outcome measures, clearly defined athlete populations, and co-produced study designs involving athletes and support teams to enhance relevance and generalisability.

## Key points

- Links between oral health and athletic performance give dentists a compelling reason to emphasise prevention, even in asymptomatic patients.
- Athletes and sports teams become relevant target groups for dental services focused on performance and recovery.
- Dentists can better motivate patients by connecting oral health to real-life outcomes like endurance, strength, and agility.
- Findings support dentists working alongside sports physicians, physiotherapists, and coaches, elevating the profession's role in holistic athlete care.

## Introduction

Sports performance is determined by a complex interaction of physical, technical, tactical, and psychological skills that are further influenced by physiological, biomechanical and cognitive factors.^1^ For most athletes, maximising performance in their specific sport is fundamental to their training, preparation, and daily routines. While the requirements for optimal performance widely differ between the range of sports, athletes typically aim to have optimal performance in competitions. In metrical sports like athletics (track and field) or swimming, it is straightforward to quantify performance by time (e.g., sprint or marathon) or distance (e.g., jumping or throwing). In other sports, such as team or aesthetic disciplines, assessing performance is more complex. In team sports, success depends on a combination of individual physical performance, sport-specific skills, and tactical execution, with team outcomes not always reflecting individual achievement. In aesthetic sports, performance evaluation can vary substantially between disciplines – for example, in equestrian dressage, artistic impression and technical precision are judged, whereas in bodybuilding, assessment focuses on physique and muscular definition – highlighting the diverse criteria that extend beyond a single peak performance on a specific date. Therefore, this paper will focus on objective measures of physical performance.

The world-class and elite athletes (tiers 4 and 5 as defined by McKay *et al.*)^2^ are typically supported by an interdisciplinary team of coaches, exercise scientists, physiotherapists, nutritionists, psychologists and sports physicians.^3^^,^^4^ In this interdisciplinary approach, sports dentistry has developed an increasingly important role, contributing to overall health and well-being.^5^^,^^6^ For instance, a feasibility study in Great Britain Cycling, Rowing and a Premiership Rugby Union team demonstrated that a simple oral health intervention, delivered to athletes and support staff, was associated with improved oral health behaviours and reduced self-reported performance impacts.^7^

Therefore, our aim is to provide a comprehensive overview of what sports performance is, how it can be measured and how oral health influences sports performance. As sports performance is a broad field, we will focus on sports-specific skills, physical and psychological factors, as well as biomechanics in the context of explosive sports, characterised by short-duration, high-intensity efforts (e.g., sprinting, weightlifting), and endurance sports, which require prolonged, submaximal exertion supported by aerobic capacity (e.g., long-distance running, cycling). Then, we will elaborate on the (possible) mechanisms and outline how practice and sports dentistry research should incorporate performance measurements to further advance the knowledge in the areas of oral health and sports performance.

## What is performance in sport and how is it measured?

### Key components of sports performance

Sports performance is complex and strongly influenced by sport-specific skills and individual physical and psychological characteristics. While the main components of sports-specific skills refer to techniques and tasks that develop over a longer period of motor learning and practising, the physical and psychological characteristics are influenced by multiple modifiable and non-modifiable (e.g., anthropometric, hormonal) factors. Modifiable physical factors can be categorised into strength, power, speed, endurance, flexibility and agility, and typically adapt to adequate training stimuli.^8^ Most muscular and cardiorespiratory adaptations are temporary and require a well-coordinated interaction of regular (overload) exercising and recovery periods as well as hydration and nutrition.^9^^,^^10^^,^^11^^,^^12^^,^^13^ Psychological factors have been linked to athletic performance, including evidence of an association between psychopathology and performance outcomes.^14^ Furthermore, self-motivation, cognitive capacity, emotional intelligence, coping skills and mindfulness interventions have been shown to influence sports performance.^15^^,^^16^^,^^17^ In the context of sports-specific performance, biomechanics can determine the role of movement efficiency, for example, how efficiently the centre of mass can be transported by the musculoskeletal system from point A to B. Biomechanics analyses the mechanical principles of movement, while the sensorimotor system provides the sensory input and motor output that enable and refine these movements, making the two intrinsically interdependent in sports performance. It also analyses the underlying (inertia, velocity-based and reactive) forces associated with muscle strength-related and coordinative aspects of movement. Accordingly, sports biomechanics considers three-dimensional aspects of movements and forces and are quantified by kinematical, kinetical and spatiotemporal parameters.^18^

### Analysing performance in metrical sport

Measuring metrical sports performance is quite straightforward as they are typically quantified by a metric number per unit. For example, in athletics, performances in all disciplines are measured in time or distance, and the respective values can even be compared with previous data, other athletes or norms (e.g., World Athletics score).^19^

However, analysing the effects of performance-related interventions (training, nutrition, materials) and underlying mechanisms of muscular, cardiorespiratory, or metabolic adaptations is more complex.^20^^,^^21^ Multiple objective and subjective (e.g., perceived exertion, fatigue or questionnaires) measures can be used and combined to analyse internal load as well as training-related changes for endurance, strength, speed and power.^22^

The spectrum of tests for endurance performance includes standard laboratory measurements such as the V̇O₂ max test on a (running, rowing, or cycling) ergometer to estimate endurance capacity or field-based tests (e.g., 20 m shuttle run test, Yo-Yo Intermittent Test).^23^^,^^24^ They can analyse subcategories of performance, such as aerobic (e.g., running economy) or anaerobic (e.g., lactate threshold, power tests) capacities.

Explosive strength and power performances can be measured through a variety of tests, including sprint and jumping tests. A standard test for muscle force capacities is the one-repetition maximum test, which is defined by the weight which can be lifted once over the full range of motion. Force-time curves of maximal or submaximal isokinetic dynamic or isometric muscle contractions can help to analyse explosive or endurance aspects of strength.^22^

Further crucial tests for athletes' performances are agility tests. These tests generally combine sprinting abilities with several change-of-direction manoeuvres and challenge the braking and generating forces of the body while maintaining momentum.^25^ These movements occur in team, racket and combat sports. The most used tests are the Agility T-Test and the Illinois Agility Test; although, a vast range of sport-specific tests can be found in the literature.^26^

## What do we know about the effect of oral health on modifiable parameters of sports performance?

### Associations between oral health and sports performance: evidence from systematic reviews and meta-analyses

A recent systematic review and meta-analysis involving 396 athletes has examined the link between athletic performance and periodontal diseases.^27^ They found moderate strength of evidence for a relationship between periodontitis and self-reported reduction in sports performance and concluded that although performance evaluations differed across studies, periodontal diseases significantly correlated with lower self-reported sports performance. In another review, Bramantoro *et al.* (2020)^28^ evaluated the relationship between oral health and objective performance tests, including athlete and non-athlete populations. Of the eleven high-quality studies included, ten reported statistically significant associations between oral health and modifiable performance outcomes (physical fitness, body balance, cardiorespiratory function, and cognitive function). Physical fitness was mainly associated with malocclusion, periodontitis, and periapical inflammation. Endodontic disease alone was not found to be associated with poor physical performance. The review supports the negative effect of poor oral health on physical fitness and performance. Ashley *et al.* (2015)^29^ investigated the oral health impact on sporting performance in athlete populations. The systematic review included 34 studies, and the methodological quality of the included studies was reported as generally low. High prevalence of oral diseases was found (dental caries 15–75%, dental erosion 36–85%, periodontal disease 15%). The negative impact of oral health or trauma on performance was reported in four studies (with range of 5−18%).

### Oral health impacts parameters of aerobic, anaerobic and neuromuscular performance

Multiple studies support the hypothesis that poor oral health may adversely affect various dimensions of sports performance. Merle *et al.* (2022) explored interactions between signs of periodontal inflammation and systemic parameters in world-class and elite German athletes (tier 4 and 5),^2^ defined as members of German youth or senior national squads. They assessed sports performance using an ergometer and maximal aerobic capacity quantified by V̇O_2_ max (Table 1 ).^30^^,^^31^^,^^32^^,^^33^^,^^34^^,^^35^^,^^36^^,^^37^^,^^38^^,^^39^^,^^40^^,^^41^^,^^42^^,^^43^^,^^44^^,^^45^^,^^46^^,^^47^^,^^48^^,^^49^^,^^50^^,^^51^^,^^52^^,^^53^ The periodontal condition affected both V̇O_2_ max values and cycling ergometer performance: Athletes with signs of periodontitis (Periodontal Screening Index[PSI] ≥3) recorded a potentially important 5.7% lower mean V̇O_2_ max value compared to those with PSI ≤2 (55.9 ± 6.7 mL/min/kg versus 59.3 ± 7.0 mL/min/kg; *p* = 0.03). A similar trend was observed in ergometer tests, where better performance was achieved in athletes with a lower level of signs of periodontal inflammation (Papilla Bleeding Index (PBI) <0.42) who consistently reached higher maximal power on the cycling ergometer (5.0 ± 0.5 W/kg versus 4.4 ± 0.3 W/kg; *p* = 0.03). Potential associations were found between performance, body composition, blood parameters, and signs of periodontal inflammation.^30^

**Table 1:** Summary of commonly used tests of modifiable factors (endurance, sprint, and power) of sports performance and their reported associations with oral health

**Test**	**Description**	**Test type**	**Evaluation with oral health**
**Endurance**			
V̇O_2_ max test	Measures maximal oxygen uptake via graded treadmill or cycle test with gas analysis^32^	Lab	Worse periodontal health associated with lower VO_2_ max and relative load output in elite and world-class athletes^30^
Lactate threshold test	Identifies the point where blood lactate rises sharply, useful for setting training zones^33^	Lab	No statistically significant association with periodontal status in elite and world-class athletes^30^
Running economy	Measures oxygen consumption at submaximal speeds; indicates efficiency^34^^,^^35^	Lab	NA
Critical speed test	Estimates sustainable high-intensity running speed using multiple time trials^36^	Field	NA
Cooper 12-minute run test	Distance covered in 12 minutes; estimates VO_2_ max^37^	Field	NA
1.5-mile run test	Estimates VO_2_ max from time taken to run 1.5 miles (2.4 km)^38^	Field	NA
Yo-Yo Intermittent Test	Assesses endurance and recovery in high-intensity intermittent sports^39^	Field	Highly-trained/national-level adolescent basketball players with poor oral hygiene presented a lower value at pre- and post-Yo-Yo Intermittent Recovery Test Level 1 compared to good oral hygiene group^40^
Time Trials (e.g., 3 km, 5 km, 10 km…)	Race-like trials that estimate race fitness and endurance capacity^41^	Field	NA
Beep test	Progressive shuttle run with increasing pace; estimates VO_2_max^42^	Field	NA
**Sprint and power**			
Linear sprint tests	Measures acceleration and/or maximal sprinting speed; can be divided into splits (e.g., 5 m, 10 m, 20 m…) and start can be performed standing or flying^43^	Field/lab	Speed test results showed that athletes with better oral health (DMFT <4) outperform those with worse oral health (DMFT ≥4) in speed tests (10-, 20-, 30 m sprints) (*p* <0.05). Success rates dropped as DMFT values increased with longer completion times^31^
Vertical jump test (CMJ, SJ), jump power	Measures lower-body power using jump height or power; often with force plate or jump mat^44^^,^^45^	Field/lab	Higher% PPD >4 was consistently correlated with lower CMJ height average in law enforcement workers^46^
Standing long jump	Measures horizontal explosive leg power,^47^ frequently used in youth^48^^,^^49^	Field	NA
Wingate anaerobic test	30-second all-out cycling test to measure anaerobic power and fatigue index^50^	Lab	Dental caries and a high DMF-T index value did not significantly affect athletic performance^51^
Isokinetic dynamometry	Measures peak torque and muscle strength across joint angles^52^	Lab	NA
Force-velocity profiling (e.g., sprint force-velocity test)	Analyses sprint mechanics to determine optimal power output^53^	Field/lab	NA
CMJ, countermovement jump;,DMFT, Decayed Missing and Filled Teeth; NA, not applicable; PPD, probing pocket depth; SJ, squat jump			

Similarly, Yapici *et al.* (2019)^31^ investigated the relationship between oral health and sporting performance in athletes (no tier stated) in Turkey. Little information was provided on the definition of athletes; although, they were reported as having a mean age of 20.4 years (± 1.3 years), training for 6–8 hours per week and actively involved in sport for 6.6 years (± 2.6 years). Oral health was evaluated by Decayed, Missing, Filled Teeth (DMFT), Significant Caries Index and Plaque Index. Sports performance included t-drill, zig-zag, lateral change of direction (LCD) and 505 tests to assess agility, as well as 10, 20 and 30 m short sprint tests to assess speed. Athletes with DMFT <4 performed better in the agility tests and speed tests than athletes with DMFT ≥4, with all tests showing statistically significant differences. Increased DMFT values were associated with extended times of agility and speed tests. A positive low to moderate correlation was found between DMFT and agility tests [t-drill test (r = 0.428), zig-zag test (r = 0.428), LCD test (r = 0.286) and 505 test (r = 0.529)], and speed tests [short sprint, 10 m (r = 0.309), 20 m (r = 0.336), 30 m (r = 0.449) (*p* <0.05)].

### Oral health impacts wellbeing, sports participation, food intake and self-reported sports performance

Several cross-sectional studies have used self-reported questionnaires to evaluate the impact of oral health on daily activities, quality of life and sports performance among athletes (Table 2).^54^^,^^55^^,^^56^^,^^57^^,^^58^^,^^59^^,^^60^^,^^61^ The studies described in Table 2 were conducted in Nigeria, Brazil, Pakistan, the Netherlands, and the UK. The most common oral conditions assessed were dental caries (DMFT), periodontal health (Basic Periodontal Examination/Community Periodontal Index of Treatment Needs), and erosive tooth wear (Basic Erosive Tooth Wear Examination). The studies included athletes from various sports, such as table tennis, lawn tennis, badminton, cricket, cycling, baseball, basketball, and hockey, including the Olympic and Paralympic Games. However, descriptions of the athlete groups and tiers were inconsistently reported across studies. Overall, the prevalence of oral health conditions was consistently high, with dental caries reported up to 63.5%^57^ and gingivitis up to 77%.^56^ Across the reviewed studies, the impact of oral health on daily activities and self-reported sports performance was noted in most studies; although, the strength of evidence is limited by the use of unvalidated tools. Wide variation was found in the tools assessing athletes' perception and psychosocial and performance impact, with some studies using unvalidated self-administered questionnaires, while only a few were based on validated tools such as the Oral Health Impact Profile-14 or Oral Impact on Daily Performance.^62^^,^^63^ The impact on daily activities and performance included pain, difficulty eating or drinking, and effects on quality of life. More direct effects on sports performance and training included challenges participating in regular training and competition, performance affected in competitions, and a reduction in training volume. However, due to methodological limitations and the use of non-validated tools, the strength of evidence remains moderate to low.

**Table 2:** Oral health impacts daily activities and self-reported sports performance

**Author (year)** **Country**	**Oral health conditions assessed**	Population, tier according to McKay *et al*. ^2^	**Self-reported sports performance** **Validated (yes/no)**	**Oral health outcomes**	**Effect on sports performance and daily activity**
Azodo (2013),^54^ Nigeria	Caries, toothache, gingival bleeding and mouth ulcer	Athletes participated in the 2011 Nigerian University Games (n = 226), tier 3	Self-administered questionnaire (no)	The prevalence of oral health problems was 28.3% (dental caries 53%). A total of 106 experienced toothache (46.9 %), gingival bleeding (36.3 %) and mouth ulcer (40.7 %)	15.9% had experienced a dental problem during competitions, with 66.7 % reporting that the dental problem affected their performance in the competition
Frontera (2011),^55^ Brazil	Orofacial injury/trauma, mouth breathing	Brazilian Basketball Confederation Championships (n = 388); tier 3	Self-administered questionnaire (no)	50% of athletes reported orofacial injuries, with dental trauma in 69.7%. The maxillary central incisors were the most affected, followed by soft tissue injuries (60.8 %), with lip injuries being the most prevalent	22.3% related that an oral problem might cause them to stop training or playing, and 66% were aware that this could affect their physical strength
Khan *et al.* (2022),^57^ Pakistan	DMFT, BPE, BEWE	Elite Pakistani athletes who competed in any sports (athletics, table tennis, lawn tennis, badminton, cricket, cycling, baseball, basketball, hockey) at college/university, at a national or international level (n = 104), tier 3 and 4	Self-reported questionnaire for oral health and psychosocial and performance impact (no)	Mean DMFT score was 2.7 ± 3.3, 63.5% had at least one carious tooth, BPE: 46.1% had gingivitis (BPE 1–2), and irreversible periodontitis (BPE score = 3–4) was present in more than a quarter of the athletes	64.4% reported difficulty with at least one daily activity due to problems in their mouth, teeth or gums. 47.1% reported difficulty eating or drinking, with 42 (40.4%), 37 (35.5%), and 38 (36.5%) reporting that oral health problems led to difficulty in relaxing, participating in sports, and smiling without embarrassment, respectively. Regression analyses revealed a significant association between BPE and impact on both daily activities and sports performance
Kragt (2019),^58^ Netherlands	DMFT, BEWE, DPSI	Dutch elite athletes eligible for the Olympic and Paralympic Games in Rio de Janeiro 2016 (n = 116); tier 4 and 5	OHIP-14 questionnaire (yes)	In 43.0% of the athletes, clinical findings were reported with 19.8% dental decay, 21.6% with gingivitis and 0.9% with periodontitis. 15 % of all athletes had a DMFT score >10, which indicates poor oral health and 43% were in need for dental treatment	3% of the athletes missed one training or competition due to their oral health. 27.3% reported oral health has impacted their quality of life. Although, 90% reported that their oral health has never affected their training, only 28.2 % of the athletes reported to have never problems with their mouth
Gallagher (2018),^56^ UK	ICDAS, BEWE, BPE	Olympic athletes (n = 352), 256 athletes on podium potential/placement programmes for the 2016 Rio Olympic Games and 96 professional athletes; tier 4 and 5	OIDP questionnaire^61^ (yes)	Caries and ETW were found in 49.1% and 41.4% of athletes, respectively. Gingival bleeding on probing or calculus (BPE 1/2) in 77.0% and pocket probing depths of ≥4 mm (BPE 3/4) in 21.6%. The odds of having caries in team sports were 2.4 times greater than endurance athletes. The odds of having erosion were two times greater in team sport than endurance sport	110 (32.0 %) athletes reported an oral health-related impact on sport performance: oral pain (29.9 %), difficulty participating in normal training and competition (9.0 %), performance affected (5.8%) and reduction in training volume (3.8%). Other impacts were difficulty with eating (34.6%), relaxing (15.1%) and smiling (17.2 %). Several oral health problems were associated with performance impacts
Needleman (2016),^59^ UK	Dental caries, BEWE, BPE, PUFA Index	Professional soccer players (n = 187); tier 4 and 5	Shortened global evaluation of impact of oral health on quality of life^61^	36.9% players had dental caries, 53% dental erosion, Gingivitis in 80% and 5% had moderate-severe irreversible periodontal disease	More than 45% of footballers were bothered by their oral health with 19.6% reporting an impact on their quality of life and 6.9% reporting an impact on training or performance. Dentine caries had a highly statistically significant association with athlete-reported impacts
Needleman (2013),^60^ UK	Dental caries, BEWE, BPE,	Olympic athletes (n = 278); tier 4 and 5	Shortened global evaluation of impact of oral health on quality of life^61^	High levels of oral disease were observed, with dental caries affecting 55% of the athletes, dental erosion in 45%, gingivitis in 76%, and periodontitis in 15%	More than 40% of athletes presenting to the clinic were bothered by their oral health with 28% reporting an impact on their quality of life and 18 % reporting an impact on training or performance
BEWE, Basic Erosive Wear Examination; BPE, basic periodontal examination; DMFT, Decayed, Missing, and Filled Teeth Index; ICDAS: International Caries Detection and Assessment System; OHIP-14, Oral Health Impact Profile-14; OHQoL, oral health-related quality of life; OIDP, oral impacts on daily performance; PUFA,:pulp, ulceration, fistula, abscess. Participant classification framework (tiers): tier 1 recreational; tier 2 trained/developmental; tier 3 highly trained, national level; tier 4 elite/international level; tier 5 world class^2^					

## What are the potential explanatory mechanisms?

### Inflammatory pathways may impact recovery and reduce performance

Periodontitis is a chronic, multifactorial inflammatory disease associated with dysbiotic plaque biofilms, characterised by progressive destruction of the tooth-supporting apparatus.^64^ Due to the chronic inflammation, patients with severe periodontitis show increased markers of systemic inflammation, including high serum levels of C-reactive protein (CRP), tumour necrosis factor-alpha (TNF-α), moderate leukocytosis and high level of cytokines such as interleukin-6 (IL-6), IL-8 and IL-1 in comparison to healthy populations.^65^^,^^66^^,^^67^

Such raised systemic inflammation impairs glycaemic control. Conversely, reducing periodontal inflammation by standard, non-surgical periodontal therapy, lowers biomarkers of systemic inflammation and improves glycaemic control.^68^ Elevated cytokines (e.g., IL-6,^69^ TNF-α,^70^ CRP^71^) from oral infections may impair muscle physiology and repair,^72^ increase muscle fatigue,^73^ increase mild cognitive impairment,^74^ and affect endurance capacity.^75^ IL-6 is also known as an important signalling molecule during exercise, being released from working muscle fibres with increased exercise duration, intensity, and muscle glycogen depletion.^76^ Chronically elevated IL-6 levels were found to increase skeletal muscle fatigability and disrupt mitochondrial content and function independent of changes in fibre type and mass.^69^ Therefore, periodontitis can result in a prolonged recovery, leading to decreased performance.

### Oral microbiome

The complexities of microbiome analysis are starting to be reported in relation to athlete performance. In relation to the gut, *Veillonella genus* has been associated with enhanced performance via lactate metabolism.^77^^,^^78^ In the mouth, limited data suggest differences in the oral microbiome between athlete and non-athlete populations; although, whether this is cause or effect is unknown.^79^ In relation to performance, attention has focused on influencing the presence of dietary nitrate-reducing bacterial species (for instance with probiotics), which can increase the efficiency of oxygen delivery and utilisation in muscle; although, the benefit is greatest in sub-elite athletes.^80^ Outside of sport, much attention has been given to the relationship between the oral microbiome and cognitive performance. Oral microbiomes associated with oral diseases such as periodontitis are associated with cognitive impairment.^81^^,^^82^^,^^83^ It is unclear if the effects of the oral microbiome on cognitive function are direct and/or mediated through inflammatory pathways. Since there are a number of distinct oral microbiomes (for instance, salivary, tongue, gingival crevice and periodontal pocket), it is important that these are fully described in studies.

### Impaired nutrition

Optimising nutrition is a key strategy to improve recovery and performance.^84^^,^^85^ Poor oral health can negatively impact nutrition, for example, mediated by pain or discomfort while eating.^86^ Dental caries, periodontitis or ulcers can lead athletes to avoid certain foods (nuts, raw vegetables, or fruits with acidity) or choose highly processed food with reduced nutritional content. This can promote an imbalanced diet with reduced recovery capacities.^87^

### Psychological factors

Self-confidence is important for optimal sports performance but may be reduced with noticeable dental issues (bleeding gums, missing teeth, bad breath).^88^ Furthermore, it has been shown that anxiety, depression, and attention deficit hyperactivity disorder may result in poor oral hygiene that could aggravate oral health and further reduce performance.^89^ In addition, chronic pain, for example, due to caries or periodontitis, can increase anxiety or reduce mood and decision-making capacity.^90^

### Sensorimotor system

**Fig. 1:**
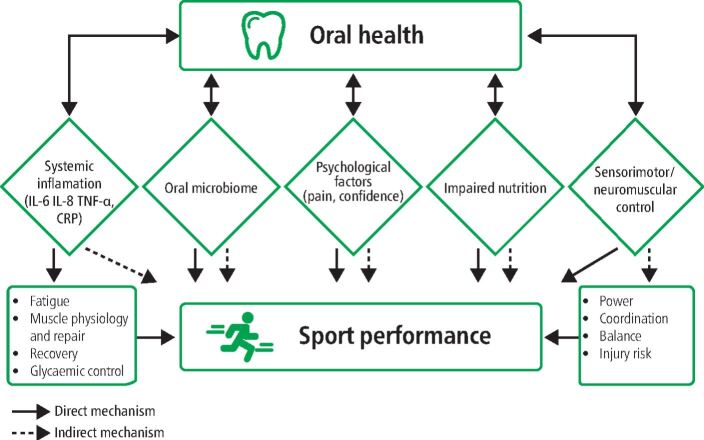
Potential mechanisms linking oral health to sports performance

The sensorimotor system integrates afferent information from different sensors from the whole body and is involved in the execution of movements. Pain is a strong input into our sensory system and dental pain may influence sensorimotor control and may influence power in consideration of the role of periodontal mechanoreceptors.^91^ Furthermore, jaw mechanics and temporomandibular disorders have been shown to affect postural stability and performance.^92^^,^^93^ The discussed potential mechanisms are depicted in Figure 1.

## Current research gaps and future directions

Future research in sports dentistry should embrace an interdisciplinary approach, integrating expertise from dentistry, sports medicine, exercise science, and public health. This collaboration is essential to better understand and address the complex interactions between oral health and sports performance. Currently, many aspects of these relationships remain unclear, highlighting the need for more high-quality research to advance the field. To achieve this, studies must employ validated and reliable outcome measures that capture both sport-specific performance indicators and comprehensive oral health status. A clear and consistent definition of the athletic population under investigation, considering factors such as sport type, competitive level, and training load, is critical for comparability and relevance. Furthermore, co-producing research with athletes, coaches, and members of athlete medical and performance support teams will help ensure that study designs and outcomes reflect real-world needs and enhance the practical impact of findings.

## Conclusion

Future efforts should focus on increasing awareness of oral health as a key component of overall athlete health, wellbeing and performance. This includes oral health screening protocols in athletes and exploring effective prevention strategies and embedding oral health education within broader athlete health promotion initiatives. Together, these directions can foster a more integrated, evidence-based approach to oral care in sports settings and may consequently further improve sports performance.
